# Regulatory Effects of a Lipid‐Lowering Strain *Lactobacillus plantarum* 58 Isolated From Dregs Vinegar on Metabolism‐Related Gene Expression, Gut Microbiota, and Metabolic Biomarkers of Hybrid Grouper Under High‐Fat Diets

**DOI:** 10.1155/anu/4888310

**Published:** 2026-05-18

**Authors:** Huizhong Shi, Liu Jiang, Yan Cai, Zihan Chen, Yongchao Hu, Yongcan Zhou, Jianlong Li, Dongdong Zhang, Zhenjie Cao, Shifeng Wang

**Affiliations:** ^1^ Collaborative Innovation Center of Marine Science and Technology, Hainan University, Haikou, China, hainu.edu.cn; ^2^ Hainan Provincial Key Laboratory for Tropical Hydrobiology and Biotechnology, School of Marine Biology and Fisheries, Hainan University, Haikou, China, hainu.edu.cn; ^3^ School of Life and Health Sciences, Hainan University, Haikou, China, hainu.edu.cn

**Keywords:** dyslipidemia, hybrid grouper aquaculture, lipid metabolism, metabolic disorders, microbiome

## Abstract

*Lactobacillus plantarum* 58 (lactic acid bacteria [LAB] 58), isolated from vinegar dregs, was incorporated at 1 × 10^8^ CFU/g diet into the feeds of hybrid groupers to assess its lipid‐lowering efficacy under normal (7.52 % lipid) or high‐fat (14.83 % lipid) regimens. A 2 × 2 factorial design generated four diets: C (normal), H (high‐fat), C58 (normal + LAB 58), and H58 (high‐fat + LAB 58). Serum biochemical parameters tests, histological section, and Oil Red O staining results indicated that in both LAB 58‐supplemented groups (C58 and H58), serum triglycerides and total cholesterol (T‐CHO) declined regardless of basal lipid content, and hepatic fat vacuolation and lipid‐droplet accumulation were markedly curtailed. The qPCR data revealed that LAB 58 elevated the levels of hepatic lipolysis genes (adipose triglyceride lipase [ATGL], carnitine palmitoyltransferase 1 [CPT‐1], farnesoid X receptor [FXR], and lipoprotein lipase [LPL]) while simultaneously suppressing lipid synthesis gene expressions (fatty acid synthase [FAS] and stearoyl‐CoA desaturase 1 [SCD‐1]) in the H58 group compared with the H group. Gut‐microbiota profiling showed a pronounced Firmicutes‐to‐Bacteroidetes shift, driven by increased Bacteroides, Faecalibacterium, and Lachnospiraceae‐UCG‐004; the relative abundance of *Lactobacillus* spp. surged, especially in the H58 group. Metabolomic analysis of intestinal contents further disclosed elevated levels of beneficial fatty acids—linoleic and α‐linolenic acids in H58 group. In conclusion, these findings demonstrate that LAB 58 alleviates lipid metabolic disorders by modulating hepatic gene expression, restructuring the gut microbiota, and enhancing beneficial metabolites.

## 1. Introduction

To reduce farming costs, aquaculture animals are often fed high‐fat diets (HFDs) [1]. However, excessive lipid intake increases the metabolic burden on the organism and disrupts the structure of the intestinal microbiota, leading to abnormal lipid absorption [1, 2]. Surplus lipids circulate and accumulate in liver tissues, inducing fatty vacuolation in hepatocytes and even irreversible inflammatory damage [1, 3]. Furthermore, increased levels of damage markers such as MDA, aspartate aminotransferase (AST), and alanine aminotransferase (ALT) provide further evidence that HFDs pose multiple health challenges to fish, including immunosuppression and antioxidant system imbalance [1].

Lactic acid bacteria (LAB), a leading source of probiotics, were often isolated from vegetables, fruits, dairy products, and fermented foods [4, 5]. In mammals, many LAB strains have been found to exhibit lipid‐lowering effects [5, 6]. For instance, *Lactobacillus plantarum* LP3 have been demonstrated to control body weight and prevent abnormal lipid accumulation of mice by adjusting the gene expressions and reshaping the intestinal microbiota structure [4]. Kimchi‐derived lactobacilli, through the cofermentation of vegetable juices, produce characteristic metabolic compounds (indole‐3‐lactic acid and phenyllactic acid), which effectively mitigated lipid droplet deposition in obese mice and human mesenchymal stem cells by modulating adipogenesis‐related signaling pathways [7]. However, in aquaculture, the functional exploration of LAB has predominantly focused on immune modulation, while their roles in lipid metabolism regulation remain systematically understudied [8]. As a commonly used probiotic in aquaculture, in‐depth studies elucidating the in vivo lipid‐reducing function and mechanisms of *Lactobacillus plantarum* have not yet been reported. Given its demonstrated beneficial effects on metabolic disorders in mammals [4, 9], we hypothesize that it may also offer a novel strategy for intervening in lipid dysregulation in fish.

The hybrid grouper (*Epinephelus lanceolatus* ♂ × *E. fuscoguttatus* ♀) is renowned for its rapid growth rate, delicious taste, and high‐quality meat, leading to immense farming volume and consumption in Southeast Asia [10]. HFD often predisposes hybrid grouper to fatty liver disease [11, 12]. To mitigate liver damage induced by HFDs in grouper, this study aimed to isolate LAB strain with lipid‐reducing activity and to elucidate their functions and underlying mechanisms in hybrid grouper. Initially, dregs vinegar, a traditional fermented food from Hainan Province, was selected for screening potential lipid‐lowering LAB due to its documented health benefits including promoting digestion, preventing aging, and reducing blood pressure and blood lipid levels [13]. Based on the results of in vitro probiotic screening experiments, *L. plantarum* LAB 58 was selected for subsequent in vivo fish feeding experiments due to its strong lipid‐lowering capacity. A feeding trial was then conducted to assess the effects of *L. plantarum* LAB 58 on hybrid grouper, specifically their growth, serum biochemical indicators (lipid and liver function parameters), liver tissue morphology, hepatic lipid metabolism gene expression, and intestinal microbiota composition under both normal diet and HFD conditions. The aim of this study was to contribute to the development of an environmentally sustainable feed additive capable of reversing the negative impacts of HFD in marine fish.

## 2. Materials and Methods

### 2.1. In Vitro Screening of Lipid‐Lowering Strains

#### 2.1.1. Isolation and Screening of Nonhemolytic LAB

One hundred twenty‐five LAB strains were isolated from of 11 dregs vinegar samples purchased from different vendors in Wenchang, Hainan Province, China. Then, hemolytic test was conducted to test the hemolysis activities of these LAB isolates. Detailed protocols are provided in Supporting Information S1.1 and S1.2. The resulting 22 nonhemolytic LAB strains were utilized in subsequent in vitro lipid‐lowering assays.

#### 2.1.2. Lipid‐Lowering Assay

##### 2.1.2.1. Cholesterol (CHO)‐Lowering Test

The CHO‐removal experiment was conducted following the methodologies outlined in previous studies [14]. This study utilized the o‐phthalaldehyde method to construct a standard curve for CHO content (see Table S1 and Figure S1 for standard curve). Simultaneously, a culture medium containing CHO‐micelles was synthesized to gauge the CHO‐removal capacity of LAB strains, using He’s method [15]. The inoculation ratio of LAB strains in the MRS‐CHOL medium was 5%, with subsequent incubation under equivalent conditions. After centrifugation at 4000 rpm for 10 min, the supernatant was measured at a wavelength of 560 nm using a spectrophotometer. The residual CHO content was detected in line with the results of the standard curve, and the CHO‐removal rate was quantified with the formula as follows:

CHO-removal rate%=100×CCHO_24h-CCHO_0h/CCHO_0h;

C_CHO_0h_: CHO content in MRS‐CHO media without LAB at 0 h;

C_CHO_24h_: CHO content in MRS‐CHO media after coincubation with LAB for 24 h.

##### 2.1.2.2. Triglyceride‐Lowering Test

With reference to previous research methods [16], sterilized MRS were mixed with the emulsion (2.0% PVA aqueous solution (Xilong Science Co., Ltd., Guangzhou, China)/vegetable oil from local supermarkets, ratio 3:1), and the mixture was fully emulsified for 20 min at a frequency of 500 W by using the ultrasonic cell crusher (Scientz, Ningbo, Zhejiang, China), resulting in a stable MRS‐TG media. Activated LAB solution was transferred into MRS‐TG media to a 3% final concentration and cultured at 37 °C for 48 h. Following 0 and 48 h of cultivation, centrifugation was performed at 3500 × g for 15 min to harvest the supernatant fluids. The triglyceride levels in the supernatants were quantified with the assay kit (A110‐1‐1) from Nanjing Jiancheng Bioengineering Institute, China, and were denoted as C_TG_0h_ and C_TG_48_, respectively. The total triglyceride (TG)‐removal rate was counted employing the subsequent formula:

Triglyceride-removal rate%=100×CTG_48h-CTG_0h/CTG_0h;

C_TG_0h_: Triglyceride content in MRS‐CHO media without LAB at 0 h;

C_TG_48h_: Triglyceride content in MRS‐CHO media after coincubation with LAB for 48 h.

LAB 58 was finally selected for more tests.

#### 2.1.3. Identification and Probiotic Characterization of LAB 58

16S rRNA sequencing method was used to identify strain LAB 58. Tolerance to gastrointestinal fluid, hydrophobicity, and aggregation rate tests were conducted to evaluate the probiotic characteristics of LAB 58. Detailed protocols for the above tests are provided in Supporting Information S1.3–S1.5 and Table S2.

### 2.2. Diet Preparation and Fish Maintenance

After in vitro screening, *L. plantarum* LAB 58 was selected for subsequent in vivo feeding experiments. Following centrifugation, the bacterial cells from activated LAB 58 cultures were collected and washed with sterile PBS solution three times, followed by resuspension until OD600 ~1. The basal feed and high‐lipid feed (with its ingredients and approximate composition outlined in Table 1) were supplemented with either LAB 58 (at a dose of 1 × 10^8^ CFU/g) or an equal volume of sterile PBS, resulting in four distinct diets: C diet (basal feed + sterile PBS solution), C58 diet (basal feed + 1 × 10^8^ CFU/g LAB 58), H diet (high‐fat feed + sterile PBS solution), and H58 diet (high‐fat feed + 1 × 10^8^ CFU/g LAB 58). Diets were prepared weekly.

**Table 1:** Ingredients and approximate composition of basal and high‐lipid feeds.

Ingredients	Basal feed	High lipid feed
Fishmeal	60%	60%
Corn gluten meal	8%	8%
Potato starch	12%	12%
Fish oil	1%	4.5%
Soybean oil	1%	4.5%
Microcrystalline cellulose	14%	7%
CMC‐Na	0.4%	0.4%
Vitamin premix^a^	0.2%	0.2%
Mineral premix^b^	1%	1%
Choline chloride	0.2%	0.2%
Ca(H_2_PO_4_)_2_	2%	2%
Butylated hydroxytoluene	0.2%	0.2%
Total	100%	100%
Nutrient level		
Moisture	12.17	14.49
Ash	8.57	8.62
Crude protein	58.86	59.34
Crude lipid	7.52	14.83

^a^: Vitamin premix (g/kg): VA: 10.00 g; VD: 350.00 g; VE: 99.00 g; VK: 35.00 g; VB_1_: 25.50 g; VB_2_: 25.00 g; VB_6_: 50.00 g; VB_12_: 0.10 g; VC, 120.00 g; Ca‐VB_5_: 61.00 g; VB_3_: 101.00 g; VB_7_; 2.50 g; VB_8_: 153.06 g; VB_9_: 6.25 g; cellulose: 411.59 g.

^b^: Mineral premix (g/kg): NaH_2_PO_4_: 80.00 g; ZnSO_4_·7H_2_O: 28.28 g; CuSO·5H_2_O: 19.84 g; KCl: 15.33 g; FeC_6_H_5_O_7_: 13.71 g; MgSO_4_·H_2_O: 12.43 g; CoCl_2_ · 6H_2_O: 4.07 g; Na_2_SeO_3_: 2.00 g; KIO_4_: 0.03 g; MnSO_4_·7H_2_O: 0.12 g.

Healthy hybrid grouper (initial weight = 10.39 ± 1.12 g and initial length = 7.6 ± 1.8 cm) was purchased from Changjiang Chengzhe Biotech Ltd. (Changjiang, China) and transferred into the fishery at Yu Hai Lanke Biotech Ltd. (Danzhou, China) for subsequent trials. During the 14‐day temporary rearing stage, commercial feed (provided by Santong Bioengineering Co., Ltd., Weifang, China) was administered twice daily (8:30–9:00 am and 5:30–6:00 pm) until satiation. In the entire experimental trials, the water environmental parameters were maintained at 28.3 ± 0.8 °C, dissolved oxygen > 6.1 mg/L, and total ammonia nitrogen < 0.22 mg/L with running mariculture. The HFDs and concentration of LAB 58 were determined based on the preliminary experimental results. Each treatment group was composed of three replicates, with a total of 30 grouper fish in each group: C group (basal diet), C58 group (C58 diet), H group (H diet), and H58 group (H58 diet). The daily feeding amount was 3% weight of hybrid grouper, which were fed twice daily in the 56‐day feeding trial. Undigested feed and waste were cleaned at 1.5 h postfeeding by a siphon.

### 2.3. Sampling

Nine hybrid groupers in each group were randomly selected and anesthetized using a 120 mg/L solution of tricaine methanesulfonate (Sigma, Gillingham, Dorset, UK). After measuring the body weight, the body surface of the hybrid grouper was fully cleaned with 75% alcohol (Sigma, Gillingham, Dorset, UK). The blood sample was collected via caudal vein puncture using a disposable 1 mL syringe and then transferred into a 1.5 mL sterile centrifugation tube for 3 h at 24 °C, followed by storage at 4 °C overnight. The prestratified blood was centrifuged (3000 rpm, 15 min, and 4 °C) to acquire the supernatant, which was stored in new sterile tubes at −80 °C for further analysis. After blood collection, the liver was excised from the hybrid grouper, weighed, and cut into small pieces. Tissue blocks were either fixed in 4 % paraformaldehyde (Biosharp, Hefei, China) or snap‐frozen in liquid nitrogen, depending on the downstream analytical requirements. The intestinal contents were gently squeezed out of the intestine with forceps and snap‐frozen in liquid nitrogen. The remaining intestinal contents were sampled for 16S rRNA sequencing and lipid‐related biomarker analysis.

### 2.4. Growth Performance and Morphological Indices

The weight of each hybrid grouper and total feed weight were measured and recorded on Day 0 and Day 56 for analyzing the weight gain rate (WGR) and feed conversion ratio (FCR). The visceral mass and liver of dissected hybrid grouper were weighted for measuring the viscerasomatic ratio (VSI) and hepatosomatic index (HIS). These above growth indexes were calculated using the following formulas:

WGR%=100×WT−WI/WI,

FCR%=WF/WT−WI,

VSI%=100×WVi/Wi,

HIS%=100×WHi/Wi.

*W*_*T*_: total terminal weight; *W*_*I*_: total initial weight; *W*_*F*_: the weight of total feed intake; *W*_Vi_: the visceral mass weight of the *i* ^th^ dissected fish; *W*_*i*_: the body weight of the *i* ^th^ dissected fish; *W*_Hi_: the liver weight of the *i* ^th^ dissected fish.

### 2.5. Serum Biochemical Indices Assay

Total cholesterol (T‐CHO), TG, high‐density lipoprotein cholesterol (HDL‐CHO), low‐density lipoprotein cholesterol (LDL‐CHO), AST, and AST activities were tested following protocols from Nanjing Jiancheng Bioengineering Institute (China, Nanjing).

### 2.6. Histopathology of Liver

For hematoxylin and eosin (H&E) staining, refer to the experimental scheme of Wang et al. [17]; liver tissue blocks fixed in 4% paraformaldehyde were processed through graded ethanol, xylene, and paraffin embedding. Sections were then dewaxed and stained with H&E following the manufacturer’s protocol (Solarbio, Beijing, China).

For Oil Red O staining, the 4% paraformaldehyde‐fixed liver blocks were rinsed with 60% isopropyl water solution for 5 min and then transferred into Oil Red O solution and stained at light‐avoiding conditions for 15 min.

The H&E and Oil Red O sections were photographed under a light microscope (Nikon, Eclipse Ci‐L, Japan).

### 2.7. qPCR

The RNA was extracted from about 100 mg liver chunks by using the Promega RNA extraction kit (Madison, WI, USA) and then reverse‐transcribed to generate cDNA. To calculate the relative mRNA expression, this newly synthesized cDNA was conducted to the following qPCR programs: first step: 3 min at 95 °C; second step: 95°C (×10 s) and 60°C (×30 s) for 40 thermal cycles; third step: 95°C (×15 s) and 60°C (×1 min) once; and fourth step: 5 s at 60°C for melting. CT values were measured by the 2^-ΔΔCt^ method with the beta‐actin expression as an internal reference. All gene primers are listed in Table 2.

**Table 2:** Primer sequences for qPCR.

Gene description	Sequence	Accession number
ATGL	F: ATTGAGCACCTTCCACCCA	XM_049574965.1
	R: CCGAATCCATCCCACATCTT	
CPT‐1	F: TCCTTACCGTGGTCCCTCT	XM_033621981.1
	R: CTTTCCATCTGCTGCTCTATCTC	
FAS	F: CGGGTGTCTACATTGGGGTG	XM_049563855.1
	R: GAATAGCGTGGAAGGCGTTT	
FXR	F: TCCTAACACCAGATCGGCCT	OK572533.1
	R: TGTTACGCCTTTCTCCTGGTG	
LPL	F: TTCAACAGCACCTCCAAAACC	XM_049587375.1
	R: GTGAGCCAGTCCACCACGAT	
SCD‐1	F: GCAGAGTTTGTAGCCGGTGT	XM_049599957.1
	R: TGCTTCTTGGGTTTGGCTCA	
SREBP‐1c	F: AGCTGTTTACACACTCCACTCG	XM_049588270.1
	R: ACTGTGGAAGCTACAGGGCA	
β‐Actin	F: TACGAGCTGCCTGACGGACA	XM_049560987.1.
	R: GGCTGTGATCTCCTTCTGC	

Abbreviations: ATGL, adipose triglyceride lipase; CPT‐1, carnitine palmitoyltransferase 1; FAS, fatty acid synthase; FXR, farnesoid X receptor; LPL, lipoprotein lipase; SCD‐1, stearoyl‐CoA desaturase 1; SREBP‐1c, sterol regulatory element‐binding protein 1c.

### 2.8. Gut Microbiome Analysis

The contents in the gut were collected after feeding within 5 h for 16S rRNA sequencing and microbiome analysis. According to the manuscript of Tiangen MiniBEST bacteria genome DNA extract kit (Tiangen, Beijing, China), the total DNA of these samples was extracted and examined. The 16S rRNA gene amplification occurred within the V3‐V4 region, and the resulting PCR products were utilized for library construction by NEXTFLEX Rapid DNA‐Seq Kit. The library was then sequenced on the Illumina MiSeq PE300 or NovaSeq PE250 platform and analyzed using the Majorbio Cloud Platform in accordance with the established protocol [18].

### 2.9. Lipid‐Related Biomarker Analysis in Intestinal Contents

Approximately 50 mg of intestinal content was taken and mixed with 400 μL of a 1:1 acetonitrile–methanol mixture, followed by vortexing for 30 s. Subsequently, the mixtures underwent 30 min of ultrasonic extraction at 4°C using a frequency of 40 kHz. After the extraction was completed, liver blocks were first allowed to stand for 30 min at −20°C, then centrifuged at 4°C (13,000 × g, 15 min). The supernatant, after being dried with nitrogen gas, was blended with 120 μL of a 1:1 acetonitrile–water mixture; then, the ultrasonic extraction and centrifugation steps were repeated as previously described. The supernatant was collected as the working solution for subsequent analysis. The sample was analyzed using an AB SCIEX company’s ultrahigh‐performance liquid chromatography‐tandem mass spectrometry (UPLC‐TripleTOF) system to determine the content of T‐CHO, linoleic acid, and glyceryl linolenate in the intestinal content. The chromatographic and mass spectrometric conditions were configured according to the parameters set by Hu et al. [19] in their study.

### 2.10. Statistical Analysis

In vitro indices, growth performance, and morphological indices were compared using one‐way ANOVA followed by post hoc tests. Gut microbiota abundance and the relative proportions of bacteria were assessed using Kruskal–Wallis and Wilcoxon tests, as well as one‐way ANOVA. Statistically significant differences in serum biological parameters, qPCR data, and Oil Red O staining areas were analyzed by SPSS software (version 23.0, Chicago) with two‐way ANOVA (*L. plantarum* LAB 58 × diet). *p*  < 0.05 represented statistically significant:  ^∗^ *p* < 0.05 (compared to the group fed a basal diet) and # *p* < 0.05 (compared to the group supplemented with LAB 58), refer to methods of Li et al. [20]. Prism 9.0 software (GraphPad, USA) and R‐studio were used for data comparison and graphical representations. All data were visualized with the mean ± standard deviation (SD).

## 3. Results

### 3.1. Screening and Probiotic Characterization of LAB 58

**Figure 1:**
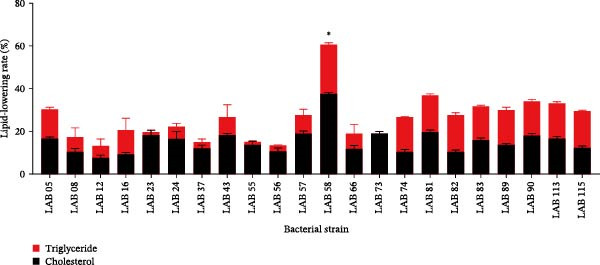
Cholesterol and triglyceride‐lowering rate of 22 potential probiotics in vitro.  ^∗^ *p* < 0.05.

After conducting hemolysis tests (Table S3), 22 LAB strains derived from dregs vinegar were preliminarily screened. The in vitro lipid‐lowering activity experiment results of 22 LAB strains were illustrated in Figure 1. LAB 58 strain exhibited the best in vitro lipid‐lowering activity, characterized by effectively reducing the levels of T‐CHO and TG in the culture medium.

LAB 58 strain was identified as *L. plantarum* based on the alignment of 16S rDNA nucleotide sequences (GenBank ID: PP951685). A series of probiotic assays showed that LAB 58 strain exhibited good gastrointestinal fluid tolerance, cell hydrophobicity, and auto‐aggregation properties shown in Figure S2.

### 3.2. Growth Metrics and Morphological Indices

Throughout the feeding experiment, the survival rate of four experimental groups remained 100%. The results indicated that probiotic supplementation with *L. plantarum* 58 or the high‐lipid diets would not make a significant difference in WGR, FCR, and HIS in each group (*p* > 0.05) (Table 3). A significant reduction in VSI was observed in the C58 group when contrasted with the other three groups ( ^∗^ *p* < 0.05). The livers in the H group were discolored, yellowish‐brown.

**Table 3:** Growth metrics and morphological indices of hybrid grouper.

Growth parameters	C	C58	H	H58
WGR/%	175.53 ± 14.37	169.07 ± 23.56	186.73 ± 24.27	181.43 ± 17.98
FCR/%	1.58 ± 0.23	1.74 ± 0.16	1.73 ± 0.21	2.03 ± 0.33
VSI/%	14.51 ± 1.34	11.70 ± 0.45 ^∗^	15.90 ± 1.07	13.80 ± 1.77
HIS/%	4.20 ± 0.75	4.01 ± 0.46	4.53 ± 0.54	3.80 ± 0.43
Survival rate/%	100	100	100	100

*Note:* C: group fed with control diet; C58: group fed with control diet supplementation with *L. plantarum* 58; H: group with HFD; H58: group fed with HFD supplementation with *L. plantarum* 58; HIS, hepatosomatic index; VSI, viscerasomatic ratio.

Abbreviations: FCR, feed conversion ratio; WGR, weight gain rate.

^∗^ *p* < 0.05.

### 3.3. Serum Biochemical Indices

**Figure 2:**
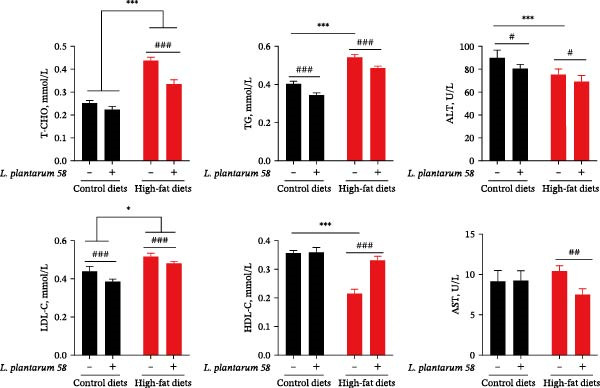
Serum lipid contents and liver function activities in the hybrid grouper fed with four types of feed.  ^∗^: *p* < 0.05,  ^∗∗∗^: *p* < 0.001, # *p* < 0.05, ## *p* < 0.01, and ### *p* < 0.001.

Serum biochemical assay results illustrated that, compared to the C group, the levels of LDL‐C ( ^∗^ *p* < 0.05), T‐CHO ( ^∗∗∗^ *p* < 0.001), and TG ( ^∗∗∗^ *p* < 0.001) in serum were significantly increased by HFD, while the amount of HDL‐C ( ^∗∗∗^ *p* < 0.001) and the activity of ALT ( ^∗∗∗^ *p*  < 0.001) were significantly decreased. Importantly, in response to stress induced by HFD, HFD‐LAB 58 hybrid grouper exhibited significantly lower T‐CHO, TG, and LDL‐C levels (### *p* < 0.001) and the functional levels of ALT (# *p* < 0.05) and AST (## *p* < 0.01) but higher HDL‐C contents (### *p* < 0.001) compared to those in the H group (Figure 2).

### 3.4. Histopathology of Liver

**Figure 3:**
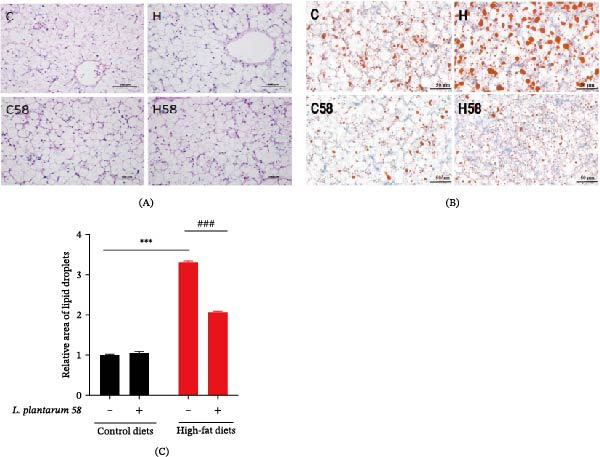
Histopathology of liver in the hybrid grouper fed with four types of feed. (A) Representative images of H&E staining (200 ×). (B) Representative images of Oil Red O staining (200 ×). (C) Quantification of the Oil Red O staining.  ^∗∗^: *p* < 0.01; ###: *p* < 0.001.

Histopathological examination revealed distinct morphological differences among the groups. As shown in H&E staining (Figure 3A), hepatocytes in groups C and C58 exhibited normal architecture with clear cellular boundaries. In contrast, group H displayed extensive intracellular vacuolation, a pathological alteration that was markedly attenuated in group H58. Moreover, Oil Red O staining and quantitative analysis (Figure 3B,C) demonstrated a significant increase in the relative area of lipid droplets in group H compared to group C ( ^∗∗^ *p* < 0.01), whereas group H58 showed a significant reduction in lipid droplet area compared to group H (### *p* < 0.001).

### 3.5. Liver Genes Pertaining to Lipid Metabolism and Their Expression

**Figure 4:**
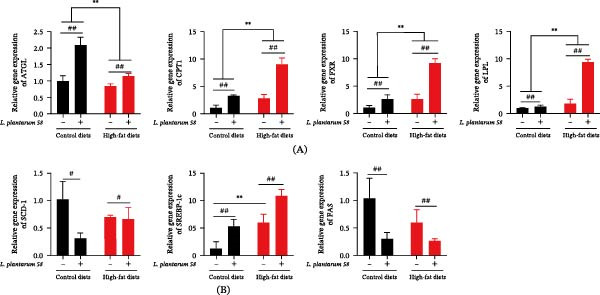
Liver lipid‐metabolism gene expression in the hybrid grouper fed with four types of feed. (A) Lipolysis‐related gene expression. (B) Lipid synthesis gene expression.  ^∗∗^: *p* < 0.01, # *p* < 0.05, and ## *p* < 0.01.

As shown in Figure 4A, HFD feeding group (Group H and Group H58) significantly altered the expression of lipid catabolism genes, upregulating carnitine palmitoyltransferase 1 (CPT‐1), lipoprotein lipase (LPL), and farnesoid X receptor (FXR) ( ^∗∗^ *p* < 0.01) while downregulating adipose triglyceride lipase (ATGL) ( ^∗∗^ *p* < 0.01) compared to normal diets group (Group C and Group C58). LAB 58 supplementation consistently upregulated these lipolysis‐associated genes in both dietary backgrounds (## *p* < 0.01). For lipid synthesis genes (Figure 4B), HFD markedly increased sterol regulatory element‐binding protein 1c (SREBP‐1c) expression ( ^∗∗^ *p* < 0.01), which was further enhanced by LAB 58 (## *p* < 0.01). Conversely, the supplementation of LAB 58 led to a significant downregulation of fatty acid synthase (FAS) and stearoyl‐CoA desaturase 1 (SCD‐1) in both the C58 and H58 groups relative to their nonsupplemented counterparts (Group C and H, respectively; # *p* < 0.05, ## *p* < 0.01).

### 3.6. Intestinal Microorganism Compositions

Gut microbiota analysis indicated that HFD significantly increased microbial richness, as evidenced by a boost in total operational taxonomic units (OTUs) and the Shannon index (Figure 5A), and this effect was reversed by LAB 58 supplementation (*p* = 0.004511). Venn diagram results indicated that Group H possessed the highest number of unique OTUs (623), followed by Group C (353), Group H58 (342), and Group C58 (328), with 196 OTUs common to all groups (Figure 5B). Beta diversity analysis revealed distinct clustering patterns: NMDS analysis showed clear separation among groups, with Group H58 significantly diverging from others due to the interactive effects of HFD and probiotic LAB 58 supplementation (Figure 5C). Hierarchical clustering results were consistent with the NMDS findings.

Figure 5Intestinal microorganism alpha and beta diversity in the hybrid grouper fed with four types of feed. (A) Five alpha diversity indices. (B) The Venn diagram. (C) NMDS graphs at the phylum and genus level. (D) Hierarchical clustering tree (phylum and genus level). (E) The pairwise comparison of intestinal differential genus analysis.

**Figure:**
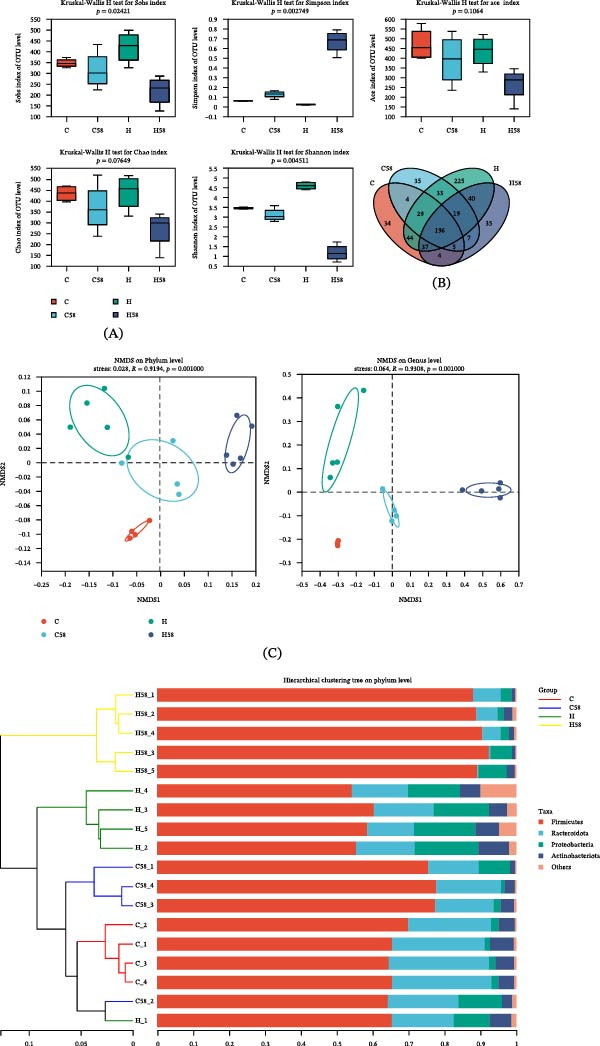

**Figure:**
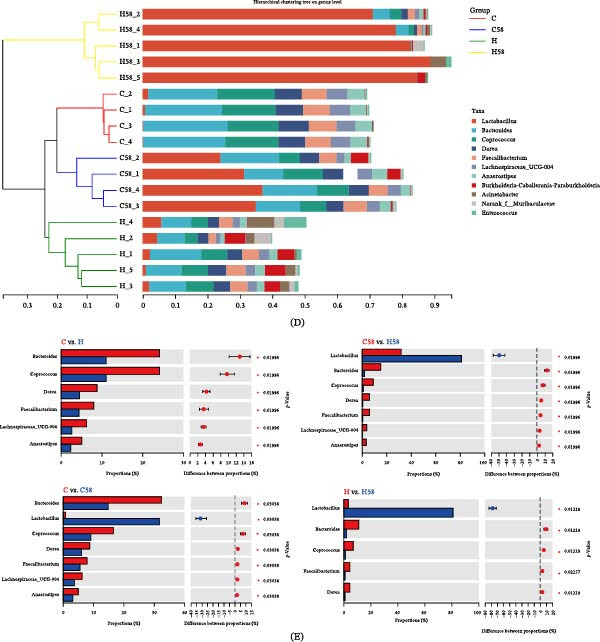

Integrating the NMDS results and the hierarchical clustering tree, pairwise comparisons (genus level) were conducted between the experimental groups to identify differentially abundant genera (Figure 5D). The relative abundances of the genera *Bacteroides*, *Coprococcus*, *Dorea*, *Faecalibacterium*, *Lachnospiraceae*‐UCG‐004, and *Anaerostipes* of Group C displayed considerable upsurge as opposed to Group H (*p* = 0.01996). The *Lactobacillus* in Group H58 displayed a marked superiority over that in Group C58 (*p* = 0.01996), occupying a dominant ecological niche, while the levels of *Bacteroides*, *Coprococcus*, *Dorea*, *Faecalibacterium*, Lachnospiraceae‐UCG‐004, and *Anaerostipes* were significantly lower in Group C58 due to differences in lipid (*p* = 0.01996). The content of the *Lactobacillus* genus was enriched in both Groups C58 and H58 (*p* = 0.03038; *p* = 0.01219), with significant reductions observed in *Bacteroides*, *Coprococcus*, *Dorea*, and *Faecalibacterium* compared to Group C (*p* = 0.03038; *p* = 0.01219) (Figure 5E).

### 3.7. Microbiological Network Analysis and Function Prediction

**Figure 6:**
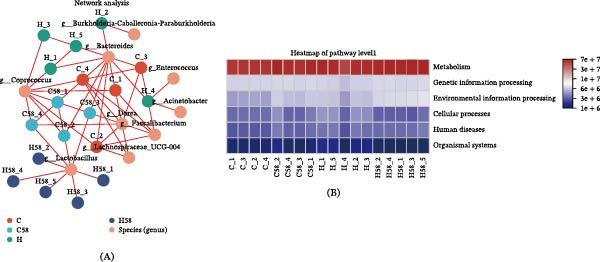
Microbiological network analysis and function prediction in the hybrid grouper fed with four types of feed. (A) Interaction network diagram between 18 samples and nine core genus. (B) Function‐related heatmap.

As illustrated in Figure 6A, the H58 group, characterized by *Lactobacillus* as its core genus, also influenced the intestinal composition of the C58 group. The genera *Bacteroides*, *Coprococcus*, *Dorea*, *Faecalibacterium*, *Lachnospiraceae*‐UCG‐004, and *Anaerostipes* played significant roles in regulating the core microbial community composition in the other three experimental groups (C, H, and C58). The functional profiling of the intestinal microbiota, as inferred from 16S rRNA gene sequencing, revealed that metabolism predominated in the functions of the gut microbiome across all experimental groups (Figure 6B). Five main metabolic pathways analysis was shown in Figure S3.

### 3.8. Lipid‐Related Biomarkers in Intestinal Contents

**Figure 7:**
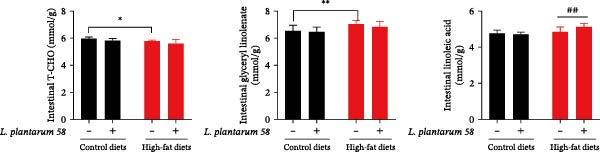
Intestinal contents lipid‐metabolism biomarkers in the hybrid grouper fed with four types of feed.  ^∗^: *p* < 0.05,  ^∗∗^: *p* < 0.01, and ## *p* < 0.01.

In Figure 7, compared to Group C, Group H’s glyceryl linolenate content was elevated ( ^∗∗^ *p* < 0.01), while the T‐CHO contents were reduced ( ^∗^ *p* < 0.05). Additionally, the supplementation of LAB 58 significantly upregulated the content of intestinal linoleic acid in HFD fed with hybrid grouper (## *p* < 0.01) compared with the H group.

## 4. Discussion

The aquaculture industry is confronted with the dual challenge of reducing production costs while mitigating the health hazards associated with HFDs [1]. Due to the current limitations in fundamental fish physiological data, research efforts are increasingly focused on developing safe and effective interventions aimed at the early disease stage of dysregulated lipid deposition [3, 11, 12]. Numerous mammalian studies have demonstrated that certain LAB‐based probiotics effectively reduce fat accumulation and enhance fat metabolism [4–7]. However, research on lipid‐lowering LAB in aquatic species is sparse: Only two zebrafish studies show *L. gasseri* M01 and *L. rhamnosus* IMC 501 cut hepatic lipids and triglycerides under HFD [21, 22]. Our study, which identifies *L. plantarum* 58 isolated from traditional Chinese vinegar dregs, is the first to investigate its hypolipidemic effects in hybrid grouper, significantly reducing liver fat and blood lipid levels.

The acquisition of probiotics against abnormal lipid accumulation initially relies on in vitro screening [23]. Previous assessments of lipid‐lowering capacity in vitro often focused solely on CHO clearance [23, 24]. However, some strains that demonstrated in vitro CHO‐lowering effects did not significantly impact in vivo lipid profiles [25]. For instance, *L. plantarum* ZY08 and *L. brevis* CAAS18052, both exhibiting strong in vitro CHO‐lowering capabilities, failed to show significant lipid‐lowering activity in mice [26, 27].. In this study, we evaluated *L. plantarum* 58 strains for in vitro lipid‐lowering activity based on both CHO and triglyceride levels to strengthen the predictive value of in vitro assays for in vivo performance. Our in vivo feeding trial results validated this dual‐parameter screening approach. Consistent with our findings, Gao and Li [28] reported that *L. acidophilus* L2‐16 significantly reduced serum triglyceride and CHO levels in mice using a similar dual‐parameter screening method.

Changes in blood biomarkers reflect an individual’s health status [29, 30]. This study demonstrated that HFD induced systemic metabolic risk, as evidenced by increased serum levels of liver function markers (AST and ALT), T‐CHO, and TG, along with histological observations of extensive hepatocellular vacuolization and marked fat droplets accumulation, which together confirmed the pathological state of aberrant lipid deposition. LAB 58 effectively reversed the abnormal lipid accumulation and liver health status induced by HFD. Specifically, the levels of LDL‐C, T‐CHO, and TG in serum were significantly lower in the H58 group compared to the H group, while HDL‐C levels were significantly higher, as well as the notable decrease in the relative area of oil‐red staining and fat vacuolation. Previous studies have also highlighted the lipid‐lowering effects of *L. plantarum*. For instance, *L. plantarum* E680 [31] and TCI 378 [32], derived from kimchi, significantly reduced CHO levels in mice, and *L. plantarum* LIP 1 [33], isolated from Mongolian horse milk, improved several lipid indices in mice. Similar hepatoprotective effects have been reported for other probiotics, including *L. paracasei* TD3, which lowered AST and ALT levels in mice [34], and *B. subtilis* Ch9, which reduced AST in carp [35].

Lipid metabolism—encompassing the synthesis and breakdown of lipids—must be tightly coordinated at the genetic level [36, 37] to maintain systemic energy balance, with the liver serving as its primary hub [22]. Key genes, such as FXR, LPL, ATGL, and CPT1, are crucial for hepatic lipid catabolism, while lipid synthesis is primarily regulated by SREBP1‐c, FAS, and SCD‐1 [36, 37]. Previous mice studies indicate that lipid overaccumulation from HFDs is associated with downregulation of lipid catabolism genes and upregulation of lipid synthesis genes in liver [38, 39]. Interestingly, our study revealed that long‐term HFDs also triggered negative feedback: Certain lipid synthesis genes (FAS and SCD‐1) were downregulated, and lipid catabolism‐related genes (CPT1, LPL, and FXR) were upregulated. Similar findings have also been observed in mice [40, 41]. However, this inherent regulatory response is often insufficient to reverse hepatic lipid overenrichment. Lipid‐lowering feed additives can effectively mitigate lipid accumulation in HFD‐fed animals by downregulating lipid synthesis genes and upregulating lipid catabolism genes. For example, 200 mg/kg pomelo polysaccharide alleviated fatty liver in hybrid grouper by reducing SREBP‐1c, FAS, and SCD‐1 expression while increasing CPT‐1 and LPL expression [42]. Similarly, 50 mg/kg berberine reduced fat synthesis in largemouth bass by downregulating FAS, SCD, and SREBP1 genes and enhancing LPL and CPT1a expression [43]. Consistent with these findings, our study showed that LAB 58 exerted lipid‐lowering effects in hybrid grouper. It significantly downregulated lipid synthesis genes (SREBP1, FAS, and SCD1) and upregulated lipid catabolism genes (FXR, ATGL, LPL, and CPT‐1) under both high‐fat and normal diets, thereby reducing hepatic lipid accumulation.

Alterations of the gut microbiota significantly contribute to the onset of fatty liver disease [44, 45]. Probiotics regulate intestinal flora by interacting with it to adjust composition, function, and metabolites [46, 47], thus emerging as a promising therapeutic strategy for balancing gut microbiota and reducing dyslipidemia risk associated with HFD [2, 47]. Notably, groups supplemented with *L. plantarum* 58 (C58 and H58) exhibited trends of increased Firmicutes and decreased Bacteroidetes, which is consistent with findings from zebrafish involving supplementatioin with *L. plantarum* SHY21‐2 and *L. plantarum* E2 [48, 49]. In our comparative analyses, both HFD and *L. plantarum* 58 supplementation significantly increased *Lactobacillus* levels in the intestinal flora of hybrid grouper. Notably, the comparison among the C58, H, and H58 groups indicated a synergistic effect: The combination of HFD and LAB 58 enhanced the proliferation of intestinal *Lactobacillus*, demonstrating that 1 + 1 > 2. The content of *Lactobacillus* in the gut of the H58 group was substantially higher than that in the H group or the C58 group, which may be attributed to the coadaptation among bacteria‐host‐feed. Martino et al. [50] demonstrated that host diet significantly shapes the symbiotic evolution of *L. plantarum* by promoting single nucleotide variants (SNVs) that enhance the strain’s ability to utilize feed components and compete in the gut. The LAB 58 strain in this study may have evolved symbiotically with the grouper, its gut flora, and the high‐fat feed, resulting in its dominance in the gut of hybrid grouper in the H58 group.

Linoleic and linolenic acids, essential polyunsaturated fatty acids, are crucial in regulating fatty acid oxidation or lipolysis [51]. Maintaining the metabolic balance of CHO is vital for cellular functions and overall health [52]. While moderate CHO addition can enhance the growth performance of aquatic organisms, excessive amounts may inhibit growth and induce liver disease [53]. Our study demonstrated that *Lactobacillus* enrichment significantly increased linoleic and linolenic acid levels in the gut while reducing CHO levels in gut contents. Elevated linolenic acid levels have been shown to promote lipid metabolism in pigeons, broilers, and ducks [54, 55]. We hypothesize that the lipid metabolism‐enhancing effect of *L. plantarum* is linked to its ability to elevate linoleic and linolenic acid levels in the body. This hypothesis is further supported by Ding et al. [4] who found that supplementation with *L. plantarum* LP3 significantly reduced CHO levels in the feces of obese mice.

## 5. Conclusion

In conclusion, *L. plantarum* 58 demonstrates significant in vitro lipid‐lowering ability, promotes lipid metabolism, reduces lipid accumulation, and enhances liver health. It achieves this through the regulation of lipid metabolism gene expression, optimization of intestinal microbiota structure, and modulation of specific metabolic markers, among other mechanisms. Therefore, the potential probiotic *L. plantarum* 58 holds great promise for improving the health of aquaculture animals. At the practical application level, it is still necessary to further explore the optimal dosage and application strategy of *L. plantarum* 58 in groupers at different developmental stages and attempt to expand its application scope, such as conducting verification experiments in a variety of farmed marine fish species.

**Nomenclature**
LAB: Lactic acid bacteria
HFD: High‐fat diets
T‐CHO: Total cholesterol
TG: Total triglyceride
HDL‐CHO: High‐density lipoprotein cholesterol
LDL‐CHO: Low‐density lipoprotein cholesterol
AST: Aspartate aminotransferase
ALT: Alanine aminotransferase
ATGL: Adipose triglyceride lipase
FAS: Fatty acid synthase
CPT‐1: Carnitine palmitoyltransferase 1
FXR: Farnesoid X receptor
LPL: Lipoprotein lipase
SCD‐1: Stearoyl‐CoA desaturase 1
SREBP‐1c: Sterol regulatory element‐binding protein 1c
OTUs: Operational taxonomic units.

## Author Contributions

**Huizhong Shi and Liu Jiang**: conceptualization, software, data curation, writing – original draft preparation. **Yan Cai**: writing – review, editing, funding acquisition. **Zihan Chen and Yongchao Hu**: methodology, software. **Yongcan Zhou**: funding acquisition, resources, writing – review. **Jianlong Li, Dongdong Zhang, and Zhenjie Cao**: software, supervision, resources. **Shifeng Wang**: conceptualization, resources, funding acquisition, editing, validation, project administration.

## Funding

This research was sponsored by the Hainan Provincial Natural Science Foundation of China (Grant 324CXTD423), the National Natural Science Foundation of China (Grant 31860739), and an earmarked fund for HNARS‐Grouper (Grant HNARS‐03‐G03).

## Disclosure

All authors have read and agreed to the published version of the manuscript.

## Ethics Statement

All fish were treated with extreme care following the guidelines set forth by Hainan University’s Animal Experimental Ethics Committee (Approval Number HNUAUCC‐2023‐00127).

## Conflicts of Interest

The authors declare no conflicts of interest.

## Supporting Information

Additional supporting information can be found online in the Supporting Information section.

## Supporting information


**Supporting Information** The reference materials can be found in the file named “Supplementary Materials.” S1.1: Isolation of LAB strains. S1.2: Hemolytic test of LAB strains. S1.3: Standard curve for cholesterol‐lowering tests (Table S1 and Figure S1). S1.4: Identification of strain LAB 58 (Table S2). S1.5: Probiotic characterization of LAB 58. S2.1: Preliminary screening results of probiotics (Table S3). S2.2: Identification and probiotic characterization of LAB 58 (Figure S2). S2.3: Five main metabolic pathways analysis (Figure S3).

## Data Availability

All data supporting the findings in this study are available within the study. All raw sequence data were uploaded to NCBI with the BioProject ID of PRJNA1129025.
