# Supplementary material for: Regulatory Effects of a Lipid‐Lowering Strain Lactobacillus plantarum 58 Isolated From Dregs Vinegar on Metabolism‐Related Gene Expression, Gut Microbiota, and Metabolic Biomarkers of Hybrid Grouper Under High‐Fat Diets
Source: Aquac Nutr. 2026 May 18;2026:4888310. doi: 10.1155/anu/4888310 (PMC13183794; doi:10.1155/anu/4888310)
Supplement: Supplementary file 1 — Supporting Information The reference materials can be found in the file named “Supplementary Materials.” S1.1: Isolation of LAB strains. S1.2: Hemolytic test of LAB strains. S1.3: Standard curve for cholesterol‐lowering tests (Table S1 and Figure S1). S1.4: Identification of strain LAB 58 (Table S2). S1.5: Probiotic characterization of LAB 58. S2.1: Preliminary screening results of probiotics (Table S3). S2.2: Identification and probiotic characterization of LAB 58 (Figure S2). S2.3: Five main metabolic pathways analysis (Figure S3). [file ANU-2026-4888310-s001.docx]

**Supplementary materials**

**1 Materials and Methods**

**1.1 Isolation of LAB strains**

Refer to the method for isolating lactic acid bacteria from fermented foods,[6] the protocol commenced with the aseptic transfer of 2 mL of each dregs vinegar sample into a sterile container pre-filled with 18 mL of aseptic saline (0.85%) using a 5 mL sterile syringe. The mixture was then thoroughly homogenized to form a uniform suspension, which served as the foundational application fluid for subsequent experimental steps. The homogenate was then diluted using a tenfold dilution method. A 0.1 mL diluted solution was spread onto MRS agar containing 1% CaCO_3_, followed by 48 h incubation at 37 °C. After the incubation period, 125 colonies with distinct, clear, and well-dispersed zones of calcium dissolution were selected and purified on MRS solid medium. The purified strains were cultured for 16 h at 37 °C, then mixed evenly with 50% glycerol for long-term storage at -80 °C.

**1.2 Hemolytic test of LAB strains**

The activated culture was streak-plated onto sheep blood agar plates, and grown at 30 °C in an inverted position for 48 hours to observe hemolysis. The hemolytic properties were ascertained using the method described by Oh and Dong, [1] and only strains exhibiting alpha- and gamma-hemolysis activities were selected for further experimental analysis.

**1.3 Standard curve for cholesterol-lowering tests**

Reaction system used for cholesterol standard curve was shown in Table S1 and the generated standard curve is shown in Figure S1.

| **Table S1**. Reaction system used for cholesterol standard curve | | | | | | |
| --- | --- | --- | --- | --- | --- | --- |
|  |  |  |  |  |  |  |
| Experiment reagent/mL | 1 | 2 | 3 | 4 | 5 | 6 |
| 0.1mg/mL Cholesterol working solution | 0 | 0.1 | 0.2 | 0.3 | 0.4 | 0.5 |
| Glacial acetic acid solution | 0.5 | 0.4 | 0.3 | 0.2 | 0.1 | 0 |
| 1mg/mL O-phthalaldehyde solution | 0.2 | 0.2 | 0.2 | 0.2 | 0.2 | 0.2 |
| Concentrated sulfuric acid solution | 4.3 | 4.3 | 4.3 | 4.3 | 4.3 | 4.3 |


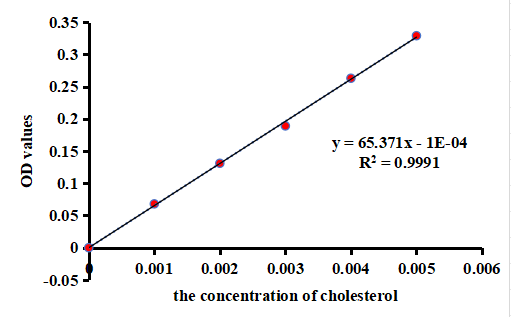


**Figure S1**. Cholesterol standard curve.

****1.4 Identification of** strain LAB 58**

The potent lipid-lowering strain LAB 58 was cultured in MRS liquid medium for 48 hours. Subsequently, DNA was extracted by following the instructions of the TianGen Bacterial Kit for subsequent gene amplification. LAB 58 strain was subjected to PCR amplification using universal primers for the 16S rRNA sequence (27F and 1492R) .[2] The 25 μL reaction system was executed according to the protocol outlined in [Table S2](#补充表2) for the PCR procedure. After verification via gel-electrophoresis, the suitable PCR products were sequenced by Tianyi Huiyuan Biotechnology Co., Ltd. (Guangzhou, China). The LAB 58 sequence was then then blasted in NCBI-Genbank database and identified as *Lactobacillus plantarum*.

**Table S2**. Reaction procedure for PCR

| Step | Temperature | Time | Cycle |
| --- | --- | --- | --- |
| Pre-denaturation | 94 ℃ | 5 min | 1 X |
| Denaturation | 94 ℃ | 30 s | 30X |
| Annealing | 56 ℃ | 30 s |  |
| Extension | 72 ℃ | 2 min |  |
| Final-extension | 72 ℃ | 10 min | 1 X |
| Storage | 4 ℃ | ∞ |  |

****1.5 P**robiotic characterization of LAB 58**

**1.5.1 Tolerance to gastrointestinal fluid test**

LAB 58’s tolerance to gastrointestinal fluids was appraised in line with Manhar’s procedure.[[3](#【32】)] 100 mL of artificial gastric juice (pH 2.0 and pH 3.0) was made with 0.5 g NaCl, 0.3 g pepsin, and 1.0 M HCl for pH adjustment, and then performing a sterilization process. The formulation for simulated intestinal juice included the addition of 2 mg of trypsin and 0.3% bovine bile salts to 20 mL of sterile PBS, with pH adjustments to 6.8 and 8.0, and subsequent filtration through a 0.22 μm filter. LAB, activated at 37 ℃, were inoculated into the artificial gastrointestinal juice at a 2% inoculum ratio and incubated for 4 h. The viable bacterial counts (log_10_CFU/mL) for each strain were ascertained by the plate-count approach.

**1.5.2 Hydrophobicity tests**

Adapting the protocol from Rosenberg with minor revisions,[4] post 48-h incubation, the LAB 58 cultures were subjected to centrifugation (3000 rpm, 10 min, 4 °C). LAB 58 pellets were then washed three times and resuspended in PBS to achieve an optical density (OD_600_) equivalent to 1.0. Subsequently, the suspension of LAB 58 was individually mixed with chloroform, toluene, and ethyl acetate, and vortexed for 2 min to ensure thorough mixing. Subsequently, the aforementioned mixture was left undisturbed at 25 °C, resulting in a clear phase separation. Finally, the LAB 58’s hydrophobicity was measured at 600 nm absorbance values.

Hydrophobicity (%) = (OD_0h_-OD_th_)/OD_0h_) × 100.

OD_0h_: OD values without organic solvent at 0 h;

OD_th_: OD values with organic solvent at t h.

**1.5.3 Aggregation tests**

In accordance with the methodology established by Aarti et al. [5] with minor procedural adaptations, after washing and resuspending in PBS, the LAB 58 solution was prepared into a homogeneous bacterial suspension with an OD_600_ of 1.0. At precise time intervals, specifically 0, 1, 2, 3, and 24 h post incubation, the supernatant of the cell suspension was diluted with PBS at a 1:9 ratio for absorbance measurement at 600 nm. The aggregation rate was calculated as follows:

Aggregation (%) = [1-(OD_th_ / OD_0h_)] ×100.

OD_0h_: OD values without organic solvent at 0 h;

OD_th_: OD values with organic solvent at t h.

**2 Results**

**2.1 Preliminary screening results of probiotics**

**Among the 125 bacterial strains, 22 LAB strains exhibiting both α- or γ-hemolytic activity were selected (Table S3). These strains were then advanced to the next stage of screening for lipid-lowering probiotics.**

****Table S3**. Evaluated LAB strains hemolysis property**

| Bacterial strain | Hemolytic test |
| --- | --- |
| LAB 05 | Gamma type |
| LAB 08 | Gamma type |
| LAB 12 | Gamma type |
| LAB 16 | Gamma type |
| LAB 23 | Gamma type |
| LAB 24 | Gamma type |
| LAB 37 | Gamma type |
| LAB 43 | Gamma type |
| LAB 55 | Alpha type |
| LAB 56 | Gamma type |
| LAB 57 | Gamma type |
| LAB 58 | Gamma type |
| LAB 66 | Gamma type |
| LAB 73 | Gamma type |
| LAB 74 | Gamma type |
| LAB 81 | Gamma type |
| LAB 82 | Gamma type |
| LAB 83 | Gamma type |
| LAB 89 | Alpha type |
| LAB 90 | Gamma type |
| LAB 113 | Alpha type |
| LAB 115 | Alpha type |

**2.2 Identification and probiotic characterization of LAB 58**

LAB 58 was subjected to a series of assays to evaluate its gastrointestinal fluid tolerance, cell hydrophobicity, and auto-aggregation properties ([Figure S](#图1)2 A-D). The results showed that after 4 hours of cultivation in simulated gastric fluid with a pH value of 3, the survival rate of LAB 58 approached 94.83%; in simulated intestinal fluid with pH values of 6.8 and 8.0, the survival rates were both above 79%. Furthermore, the hydrophobic activities of LAB 58 towards ethyl acetate, chloroform, and xylene were 41.49%, 52.91%, and 62.17%, respectively, and the auto-aggregation activity reached 64.86% within 24 h. Considering these beneficial viability indicators, LAB 58 is selected as a candidate strain for subsequent *in vivo* feeding experiments.

**
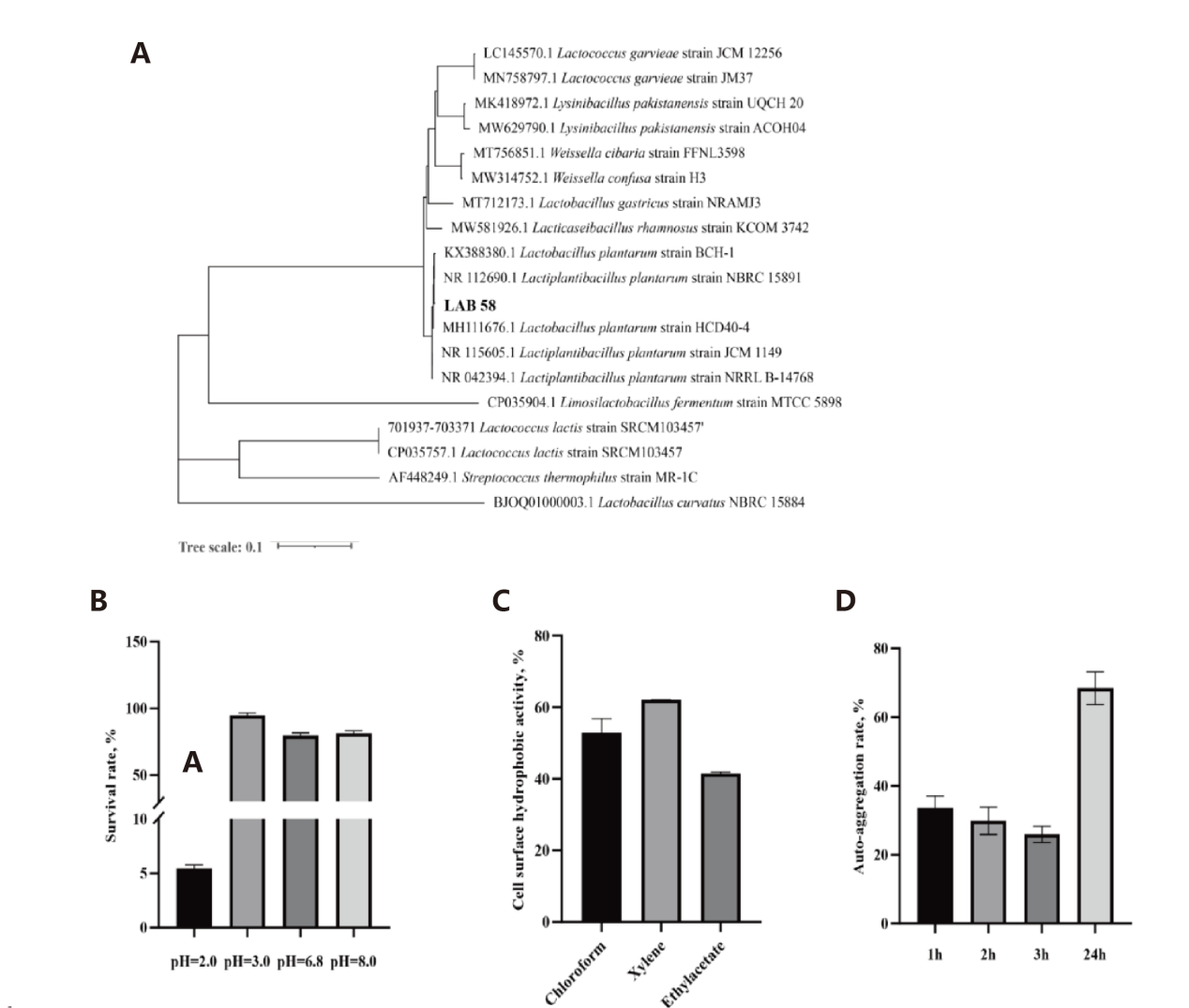
**

**Figure S2**. Identification and probiotic characterization of LAB 58. (A): a neighbor-joining phylogenetic-tree of LAB 58 based on 16S rRNA gene; (B): gastrointestinal fluid tolerance of LAB 58; (C): cell surface hydrophobicity of LAB 58; (D): auto-aggregation of LAB 58.

**2.3 Five metabolic pathways analysis**

Notably, variations in specific metabolic pathways were observed among the groups. In comparison to the groups without strain 58 supplementation (C and H groups), those receiving strain LAB 58 (C58 and H58 groups) exhibited significant enrichment in carbohydrate metabolism (##p < 0.01) and nucleotide metabolism (#p < 0.05) (Figure S3). Conversely, other three metabolic pathways did not show significant differences (*p* > 0.05) (Figure S3).


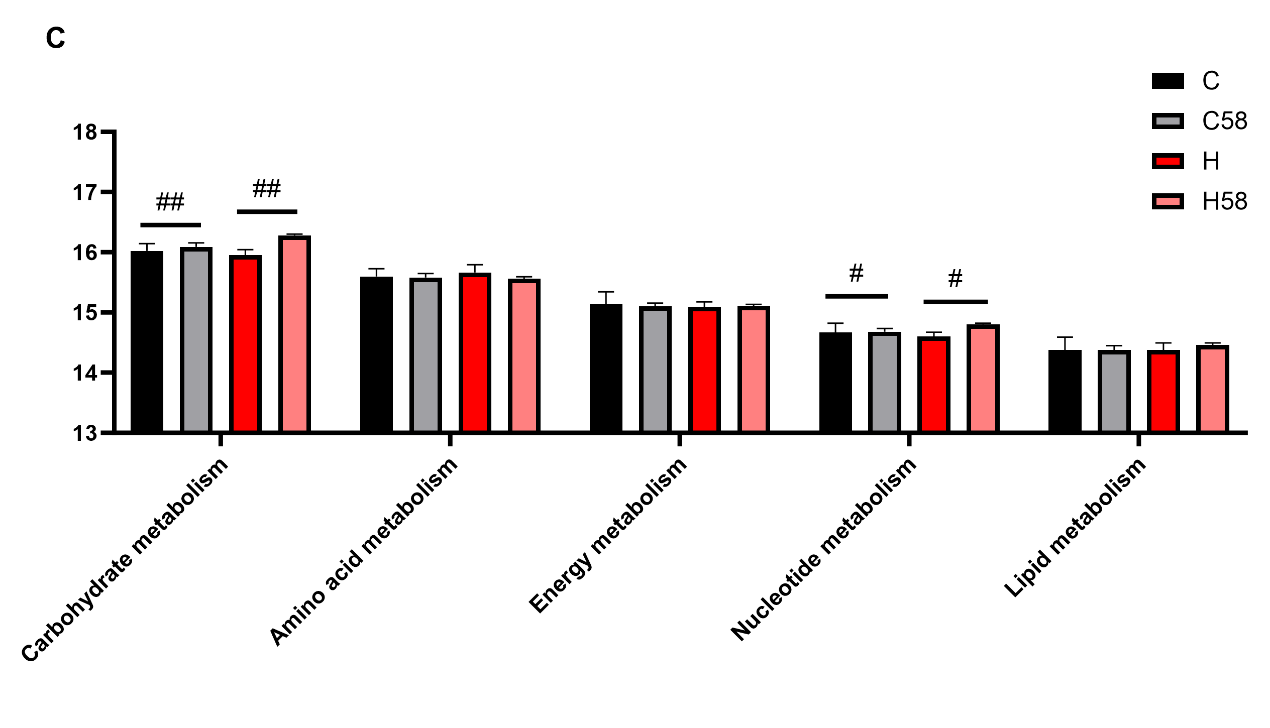


**Figure S3.** Five metabolic pathways analysis in the hybrid grouper fed with four types of feed.

#: *p* < 0.05; ##: *p* < 0.01.

**Reference**

1. Oh Y. J. and Dong S. J., “Evaluation of Probiotic Properties of *Lactobacillus* and *Pediococcus* strains Isolated from Omegisool, a Traditionally Fermented Millet Alcoholic Beverage in Korea,” *LWT-Food Science and Technology* 63 (2015): 437-444.
2. Weisburg, W. G., Barns, S. M., Pelletier, D. A., & Lane, D. J., “16S Ribosomal DNA Amplification for Phylogenetic Study,” *Journal of bacteriology* 173 (1991): 697-703.
3. Manhar A. K., Saikia D., Bashir Y., Mech R. K., Nath D., Konwar B. K., et al., “*In vitro* Evaluation of Celluloytic *Bacillus amyloliquefaciens* AMS1 Isolated from Traditional Fermented Soybean (*Churpi*) as an Animal Probiotic,” *Research in Veterinary Science* 99 (2015): 149-156.
4. Meyer E. E., Rosenberg K. J., and Israelachvili J., “Recent Progress in Understanding Hydrophobic Interactions,” *Proceedings of the National Academy of Sciences of the United States of America* 103 (2006): 15739-15746.
5. Aarti C., Khusro A., Varghese R., Arasu M. V., Agastian P., Al-Dhabi N. A., et al., “*In vitro* Studies on Probiotic and Antioxidant Properties of *Lactobacillus brevis* strain LAP2 Isolated from Hentak, a Fermented Fish Product of North-East India,” *LWT - Food Science and Technology* 86 (2017): 438-446.
6. Yu J., Gao W., Qing M., Sun Z., Wang W., Liu W., et al., “Identification and characterization of lactic acid bacteria isolated from traditional pickles in Sichuan, China,” *The Journal of general and applied microbiology* 58 (2012), 163-172.
